# Low level of stromal lectin‐like oxidized LDL receptor 1 and CD8 ^+^ cytotoxic T‐lymphocytes indicate poor prognosis of colorectal cancer

**DOI:** 10.1002/cnr2.1364

**Published:** 2021-03-06

**Authors:** Chika Katayama, Takehiko Yokobori, Naoya Ozawa, Kunihiko Suga, Takuya Shiraishi, Takuhisa Okada, Katsuya Osone, Ryuji Katoh, Toshinaga Suto, Yoko Motegi, Hiroomi Ogawa, Akihiko Sano, Makoto Sakai, Makoto Sohda, Bilguun Erkhem‐Ochir, Navchaa Gombodorj, Ayaka Katayama, Tetsunari Oyama, Ken Shirabe, Hiroyuki Kuwano, Hiroshi Saeki

**Affiliations:** ^1^ Department of General Surgical Science, Graduate School of Medicine Gunma University Maebashi Japan; ^2^ Division of Integrated Oncology Research Gunma University Initiative for Advanced Research (GIAR) Maebashi Japan; ^3^ Department of Radiation Oncology National Cancer Center Ulaanbaatar Mongolia; ^4^ Department of Diagnostic Pathology Gunma University Graduate School of Medicine Maebashi Japan

**Keywords:** CD8^+^ T‐lymphocytes, colorectal neoplasms, LOX‐1, macrophage, myeloid‐derived suppressor cells, prognosis

## Abstract

**Background:**

Lectin‐like oxidized LDL receptor‐1 (LOX‐1) has been identified as a new marker for functional myeloid‐derived suppressor cells (MDSCs) that exhibit an immunosuppressive phenotype in the tumor microenvironment (TME). However, the role of LOX‐1^+^ cells in the TME of colorectal cancer (CRC) remains unknown.

**Aim:**

This study aimed to determine the expression and significance of LOX‐1 in the TME of clinical CRC specimens.

**Methods and results:**

We performed immunohistochemical and genetic analyses of LOX‐1, CD8, *KRAS*, and *BRAF* in 128 resected CRC specimens and determined the expression of IFN‐γ and IL‐10 using real‐time reverse transcription‐polymerase chain reaction. We analyzed the correlation between LOX‐1, TME factors, gene alteration, clinicopathological factors, and disease prognosis. The co‐expression pattern of LOX‐1, hematopoietic markers, and a fibroblast marker was evaluated using multiplex immunofluorescence staining. Low stromal LOX‐1 expression and low intratumoral CD8^+^ cytotoxic T‐lymphocyte (CTL) status correlated with poor prognosis. Moreover, stromal LOX‐1‐low/CD8^+^ CTL‐low status was the most important independent prognostic factor of poor overall survival. Most of the LOX‐1^+^ stromal cells were positive for CD163^+^, indicating they were CD163^+^ M2 macrophages.

**Conclusions:**

The MDSC marker, LOX‐1, was mainly expressed by M2 macrophages in CRC tissues. LOX‐1^+^ macrophages and CD8^+^ CTLs may serve as useful biomarkers for predicting the prognosis of CRC.

## 1 INTRODUCTION

Colorectal cancer (CRC) is the third most common cancer worldwide.^1^ The tumor tissue is composed of various cells, including tumor and stromal cells, such as vascular endothelial cells, fibroblasts, inflammatory cells, and immune cells. These cells constitute a complex tumor microenvironment (TME) that facilitates tumor progression, leading to the poor prognosis of CRC.^2^ To improve the prognoses of CRC patients, we need to understand the biological significance of the TME, including multiple immune cells present in clinical CRC tissues.

Myeloid‐derived suppressor cells (MDSCs) are crucial players in immunosuppression in TME.^3^, ^4^, ^5^, ^6^ MDSCs produce immunosuppressive cytokines, such as IL‐10 and TGF‐β. Moreover, MDSCs inhibit IFN‐γ production and proliferation of CD8^+^ cytotoxic T‐lymphocytes (CTLs) and induce the immunosuppressive effects of regulatory T‐cells against cancer.^4^, ^7^, ^8^ Human MDSCs are characterized by the expression of surface markers, such as CD11b^+^ and CD15^+^.^9^ However, these markers are also expressed in other immune cells; thus, it is difficult to detect MDSCs using these markers alone.^4^ CTLs are crucial players in antitumor immunity owing to their capacity to kill tumor cells in the TME. High intratumoral density of CD8^+^ CTLs is a good prognostic factor for CRC.^10^, ^11^ Moreover, macrophages in the TME play contradictory roles in tumor immunity, that is, tumor preventing (M1 macrophages) and promoting (M2 macrophages) activities.^12^, ^13^

Lectin‐like oxidized LDL receptor 1 (LOX‐1), the main oxidized low‐density lipoprotein receptor, is involved in inflammation, atherosclerosis, and reactive oxygen species‐ and metabolic disorder‐mediated carcinogenesis.^14^ LOX‐1 is expressed in endothelial cells, smooth muscle cells, macrophages, and tumor cells, including CRC cells.^14^, ^15^ LOX‐1 was identified as a specific marker for functional MDSCs using flow cytometry and immunohistochemical analysis.^16^ Overexpression of LOX‐1 induces the differentiation of macrophages into the M2 phenotype.^17^ Tumoral LOX‐1 increases during transition from normal to neoplastic phenotype in colon adenomas; hence, the expression of LOX‐1 is associated with the early stage of the disease.^18^ To date, there have been no detailed studies focused on LOX‐1^+^ cells in the TME of CRC.

Here, we investigated the significance of LOX‐1 expression in tumor‐infiltrating immune cells using clinical CRC specimens. We examined the correlation between LOX‐1 expression and TME factors, including cytokines and CD8^+^ CTLs, changes in CRC‐related gene expression, and clinicopathological factors in CRC samples. Moreover, we analyzed correlation between stromal LOX‐1 expression, hematopoietic cells, and cancer‐associated fibroblasts. The findings of this study revealed the significance of immunohistochemical and spatial detection of LOX‐1 expression in the TME of clinical CRC tissues.

## 2 METHODS

### 2.1 Patient cohort

We enrolled 128 patients with CRC who underwent surgical resection at Gunma University hospital, Maebashi, Gunma, Japan between January 1999 and December 2009. The patients were subjected to standard surgical treatment. One hundred and two CRC patients were not administered preoperative therapy. Among the remaining 26 rectal cancer patients, 18 patients did not receive preoperative therapy, while three and five patients were administered radiation and chemotherapy, respectively before surgery. Seventeen patients had stage IV disease that was deemed surgically unresectable. Thirty‐one patients received postoperative 5‐fluorouracil (5‐FU)‐based adjuvant chemotherapy. The cohort comprised 80 males and 48 females, aged 24 to 84 years. Tumor‐node‐metastasis (TNM 7th edition) stage was 0, I, II, III, and IV in 3, 15, 35, 46, and 29 patients, respectively. Postoperative survival was measured from the day of the surgery. The median follow‐up period was 5.5 years (range: 29 days‐25.7 years).

### 2.2 Sample collection and preparation

The resected CRC specimens were fixed using 10% formaldehyde, embedded in paraffin blocks, and processed as described below. The cancerous tissues were excised and transferred to RNase‐free microtubes and immediately frozen in liquid nitrogen and stored at −80°C until RNA extraction.

### 2.3 Immunohistochemistry

Briefly, 4‐μm thick sections were cut from paraffin blocks. The sections were stained using the following primary antibodies: rabbit polyclonal anti‐LOX‐1 (ab126538, 1:200; Abcam, Cambridge, UK), mouse monoclonal anti‐CD8 (clone C8/144B, 1:50; Dako, Glostrup, Denmark), and mouse monoclonal anti‐β‐catenin (14/Beta‐Catenin, 1:200; BD Biosciences, New Jersey, USA). For immunohistochemistry, the sections immobilized on slides were deparaffinized using xylene and soaked in 0.3% H_2_O_2_/methanol for 30 minutes at 20°C to 25°C to block endogenous peroxidase activity. Antigen retrieval for CD8 and β‐catenin was performed by boiling the section‐containing slides in 0.01 M citrate buffer (pH 6.0) at 98°C for 30 minutes. For LOX‐1, antigens were retrieved upon heating the samples in a microwave oven (121°C) for 5 minutes in 0.01 M citrate buffer (pH 6.0) containing 0.01 M EDTA (pH 8.0). The sections were incubated overnight with the primary antibodies at 4°C. Histofine Simple Stain MAX‐PO kit (Nichirei, Tokyo, Japan) was used to incubate the samples with the secondary antibody for 30 minutes at room temperature. The immune reaction was captured using 3,3′‐diaminobenzidine tetrahydrochloride (Dojindo, Kumamoto, Japan). The sections were counterstained with Mayer's hematoxylin and mounted. In negative controls, the primary antibody was omitted.

### 2.4 Immunohistochemistry

Each tissue section was evaluated in a blinded fashion by at least two investigators (including one pathologist). In case of discrepancies, both investigators analyzed the slides till they reached a consensus. All sections were examined under a BX43 light microscope (Olympus, Tokyo, Japan).

LOX‐1 is expressed on tumor cells^19^ and stromal cells^16^; therefore, we defined the LOX‐1 expressed in tumor and stromal cells as tumoral LOX‐1 and stromal LOX‐1, respectively. Tumoral LOX‐1 in CRC tissues was determined using the LOX‐1 score calculated based on a semi‐quantitative assessment of the presence of LOX‐1 and its intensity in accordance with the methodology prescribed in a previous report.^18^ Briefly, the intensity of LOX‐1 staining was scored as negative/weak (0), moderate (1), and strong (2). LOX‐1^+^ cells were scored in the following manner: <10% LOX‐1 expression (0), 10% to 25% LOX‐1 expression (1), and >26% (2) LOX‐1 expression. The final scores were obtained by adding both the individual scores. According to the final score, the patients were divided into two groups, that is, low (score 0‐3) and high (score 4) tumoral LOX‐1 groups.

The number of stromal LOX‐1^+^ immune cells and intratumoral CD8^+^ CTLs was counted in selected five hotspots, using light microscopy (400× magnification; 0.058 mm^2^ field area), and density was calculated by dividing the number of positive cells by the area (cells/mm^2^). Patients were divided into two groups based on the median level of density, high (stromal LOX‐1‐H, >534.4/mm^2^) and low (stromal LOX‐1‐L, ≤534.4/mm^2^) LOX‐1, and high (CD8 + CTL‐H, >103.4/mm^2^) and low (CD8 + CTL‐L, ≤103.4/mm^2^) CD8 + CTL.

β‐catenin staining was determined according to the percentage of positively stained nuclei in 200 tumor cells. The specimens were classified as positive when there was ≥10% nuclear staining of tumor cells.

### 2.5 Total RNA extraction, cDNA synthesis, and real‐time reverse transcription‐polymerase chain reaction

Total RNA from tissue samples was extracted using the miRNeasy Mini kit (Qiagen). Eighty‐eight samples were used to synthesize cDNA using the PrimeScript RT kit with gDNA Eraser (TaKaRa Bio, Shiga, Japan). Primers for IFN‐γ and IL‐10 are as follows^20^, ^21^: IFN‐γ forward primer 5′‐TCGGTAACTGACTTGAATGTCCA‐3′, and reverse primer 5′‐TCGCTTCCCTGTTTTAGCTGC‐3′; IL‐10 forward primer 5′‐AGGGAGCCCCTTTGATGAT‐3′, and reverse primer 5′‐GGTTGGGGAATGAGGTTAGG‐3′. Real‐time reverse transcription‐polymerase chain reaction (RT‐PCR) was performed using the LightCycler 480 (Roche, Basel, Switzerland). The PCR (10 μL) included 20 ng of cDNA, 0.45 μM of each primer, and 1× PowerUp SYBR Green Master Mix (Applied Biosystems, Foster City, California). The reactions were performed in 96‐well optical plates. The following reaction cycle was used: at 95°C for 10 minutes followed by 45 cycles at 95°C for 15 seconds and 60°C for 10 minutes. The expression of IFN‐γ and IL‐10 was normalized to that of β‐actin and was analyzed using the 2^−ΔΔCT^ method.

### 2.6 High‐resolution melt curve analysis

We screened the mutations using high‐resolution melt curve analysis. Primers for exons 2, 3, and 4 of *KRAS* and *BRAF* were adapted from published studies.^22^, ^23^ PCR was performed using the LightCycler 480 (Roche, Basel, Switzerland) in a 20 μL reaction volume containing 20 ng cDNA, 0.2 μM of each primer, 2.5 mM MgCl_2_, and 1× High‐Resolution Melting Master Mix (Roche, Basel, Switzerland). The PCR conditions were as follows: preincubation at 95°C for 10 minutes followed by 45 cycles of denaturation for 10 seconds at 95°C, annealing for 10 seconds at 58°C, and extension for 10 seconds at 72°C. Melt curve analysis was performed at a range of 65°C to95°C, ramp of 0.02°C/s, and 25 acquisitions per degree. Samples were clustered into wild‐type and mutant groups using the LightCycler 480 Gene Scanning Software.

### 2.7 Multiplex immunofluorescence

We performed tyramide signal amplification labeling with the Opal reagents (PerkinElmer, Waltham, Massachusetts) using the Opal 4‐color automation IHC method. The primary antibodies used were as follows; LOX‐1 (ab126538, 1:800, Abcam, Cambridge, UK), CD15 (Carb‐3, 1:400, Dako, Glostrup, Denmark), CD11b (ab52478, 1:400, Abcam, Cambridge, UK), CD45 (1:400, #13917, Cell Signaling Technology, Inc., Danvers, Massachusetts), CD3 (ab16669, 1:300, Abcam, Cambridge, UK), CD20 (L26, 1:400, Dako, Glostrup, Denmark), CD163 (1:1000, #93498, Cell Signaling Technology, Inc., Danvers, Massachusetts), and α‐SMA (M0851, 1:100, Dako, Glostrup, Denmark). Antigens were retrieved from the tissue sections using ImmunoSaver (Nisshin EM, Tokyo, Japan). Tissue sections were incubated with fluorophores Opal 520, 570, and 690 for 10 minutes at room temperature. Antigen retrieval was performed using 10 mM sodium citrate buffer pH 6 in a microwave for 15 minutes. Finally, all sections were counterstained with DAPI. Images were captured using the BZ‐X700 microscope (Keyence). We analyzed 10 cases from the stromal LOX‐1 high group using triple‐labeled high‐power fields.

### 2.8 Statistical analysis

The *χ* ^2^ or Fisher's exact test was used to evaluate the correlation between target protein expression and clinicopathological features. Correlations were analyzed using nonparametric Spearman's rank tests and Kaplan‐Meier curves, and the log‐rank test was used for survival analysis. Overall survival (OS) was defined as the time from surgery to death due to any cause. Univariate and multivariate regression analyses were performed using the Cox proportional hazards model. Factors showing prognostic significance in the univariate analysis (*P* < .1) were employed in the multivariate Cox regression model. All statistical analyses were performed using EZR (Saitama Medical Center Jichi Medical University; http://www.jichi.ac.jp/saitama-sct/SaitamaHP.files/statmed.html).^24^ *P* < .05 was considered significant.

## 3 RESULTS

### 3.1 Distribution of LOX‐1 expression and CD8 ^+^ CTLs in CRC tissues

**FIGURE 1:**
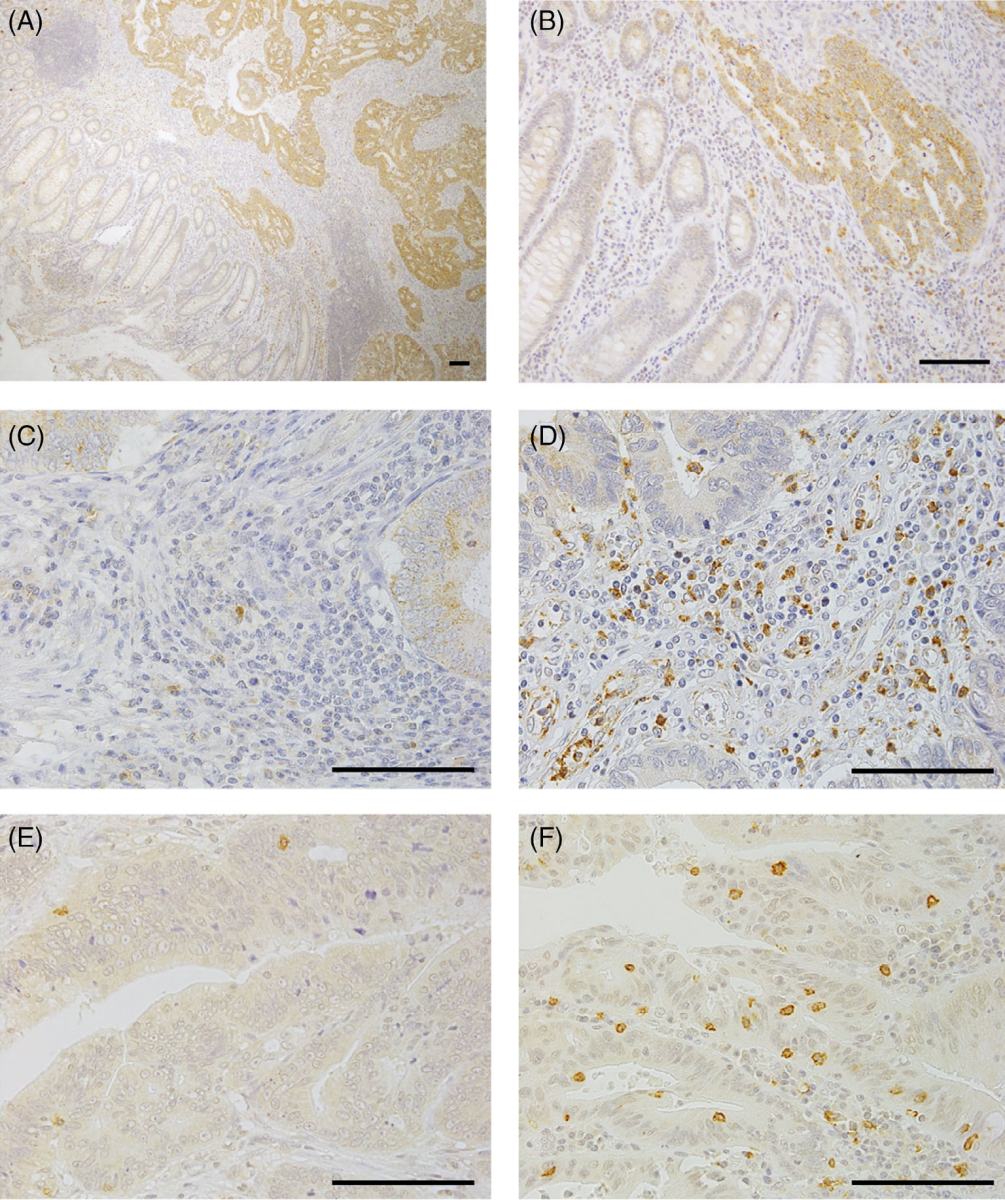
Immunohistochemistry for LOX‐1 and CD8 in CRC tissues. A and B, LOX‐1^+^ cells were localized in the tumor and stroma (A, magnification ×40; B, ×200). C, Low expression of LOX‐1. D, High expression of LOX‐1. E, Low density of CD8^+^ CTL. F, High density of CD8^+^ CTL. Scale bar = 100 μm. CRC, clinical colorectal cancer; LOX‐1, lectin‐like oxidized low‐density lipoprotein receptor‐1; CD8^+^ CTL, CD8+ cytotoxic T‐lymphocytes

CRC patient specimens (n = 128) were subjected to immunohistochemistry. Figure 1 shows representative images of LOX‐1 and CD8 expression in CRC tissue. LOX‐1 was detected in the cytoplasm of both tumor and stromal cells, with LOX‐1 expression being higher in CRC cells than that in noncancerous tissues (Figure 1A,B). High tumoral LOX‐1 expression (tumoral LOX‐1‐H group) was identified in 39.1% (50/128) of the CRC specimens, while low tumoral LOX‐1 expression (tumoral LOX‐1‐L group) was identified in 60.9% (78/128) of the specimens. The density of stromal LOX‐1^+^ cells in the tumor tissues was determined, and the median number was found to be 534.4 cells/mm^2^ (range: 0‐5.4 × 10^3^ cells/mm^2^). Based on LOX‐1 expression, stromal LOX‐1^+^ cells were divided into two groups, that is, low‐expressing stromal LOX‐1‐L group (51.6%; 66/128) and high‐expressing stromal LOX‐1‐H group (48.4%; 62/128; Figure 1C,D).

**FIGURE 2:**
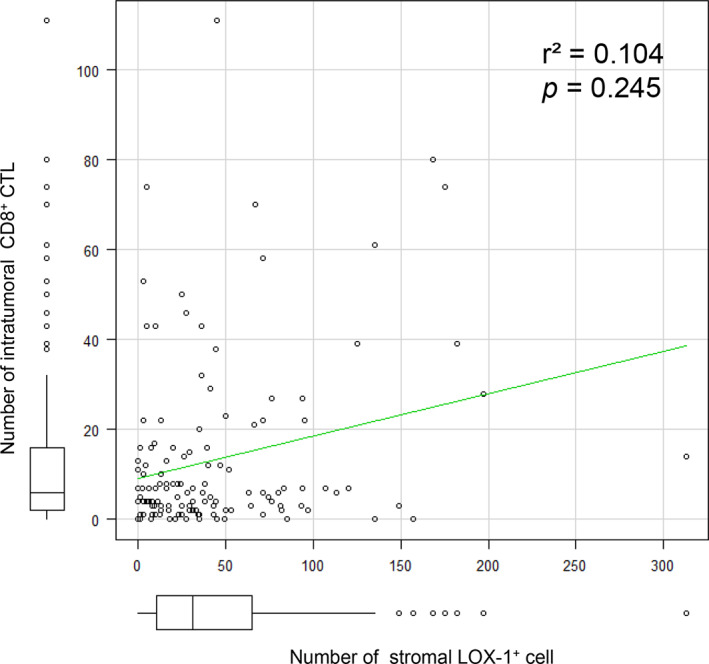
Correlation between LOX‐1^+^ cells and CD8^+^ CTLs in the tumor tissue. The graph shows the number of LOX‐1^+^ vs intratumoral CD8^+^ cells in a 0.058 mm^2^ field area. No correlation was observed between them (*r* ^2^ = .104, *P* = .245). LOX‐1; lectin‐like oxidized low‐density lipoprotein receptor‐1. CD8^+^ CTL; CD8^+^ cytotoxic T‐lymphocytes

We also counted the CD8^+^ CTLs in the tumor tissues, and the median number of CD8^+^ CTLs was 103.4 cells/mm^2^ (range: 0‐1.9 × 10^3^ cells/mm^2^). The specimens were divided into two groups based on the density of CD8^+^ CTLs. Accordingly, 52.3% (67/128) of the specimens were categorized into CD8^+^ CTL‐L group and 47.7% (61/128) were categorized into CD8^+^ CTL‐H group (Figure 1E,F). The number of stromal LOX‐1^+^ cells did not correlate with the number of CD8^+^ CTLs in this CRC cohort (Figure 2).

### 3.2 Correlation between LOX‐1 and CD8 expression and clinicopathological features

The clinicopathological significance of LOX‐1 and CD8 expression in the 128 CRC patients is summarized in Table 1. Tumoral LOX‐1 and stromal LOX‐1 status correlated with venous invasion (*P* = .043) and lymph node metastasis (*P* = .046), respectively. The CD8^+^ CTL status did not significantly correlate with the clinicopathological characteristics in either the high or low groups.

**TABLE 1:** Patient characteristics according to LOX‐1 and CD8 status in 128 CRC patients

	Tumoral LOX‐1 score			Stromal LOX‐1 status			CD8^+^ CTL status		
	Low	High		Low	High		Low	High	
Factors	n = 78 (%)	n = 50 (%)	*P* value	n = 66 (%)	n = 62 (%)	*P* value	n = 67 (%)	n = 61 (%)	*P* value
*Sex*									
Female	33 (42.3)	15 (30.0)	.192	25 (37.9)	23 (37.1)	1	24 (35.8)	24 (39.3)	.717
Male	45 (57.7)	35 (70.0)		41 (62.1)	39 (62.9)		43 (64.2)	37 (60.7)	
*Age*									
≤70	50 (64.1)	36 (72.0)	.441	42 (63.6)	44 (71.0)	.452	44 (65.7)	42 (68.9)	.711
>70	28 (35.9)	14 (28.0)		24 (36.4)	18 (29.0)		23 (34.3)	19 (31.1)	
*T stage*									
T0, T1	9 (11.5)	4 (8.0)	.901	8 (12.1)	5 (8.1)	.102	7 (10.4)	6 (9.8)	.6
T2	9 (11.5)	5 (10.0)		4 (6.1)	10 (16.1)		6 (9.0)	8 (13.1)	
T3	43 (55.1)	31 (62.0)		36 (54.5)	38 (61.3)		37 (55.2)	37 (60.7)	
T4	17 (21.8)	10 (20.0)		18 (27.3)	9 (14.5)		17 (25.4)	10 (16.4)	
*N stage*									
N0	39 (50.0)	23 (46.0)	.854	33 (50)	29 (46.8)	.046*	26 (38.8)	36 (59.0)	.066
N1	27 (34.6)	20 (40.0)		19 (28.8)	28 (45.2)		30 (44.8)	17 (27.9)	
N2	12 (15.4)	7 (14.0)		14 (21.2)	5 (8.1)		11 (16.4)	8 (13.1)	
*M stage*									
M0	62 (79.5)	37 (74.0)	.52	50 (75.8)	49 (79.0)	.679	49 (73.1)	50 (82.0)	.292
M1	16 (20.5)	13 (26.0)		16 (24.2)	13 (21.0)		18 (26.9)	11 (18.0)	
*TNM stage*									
0	3 (3.8)	0 (0)	.762	3 (4.5)	0 (0)	.498	1 (1.5)	2 (3.3)	.084
I	9 (11.5)	6 (12.0)		7 (10.6)	8 (12.9)		6 (9.0)	9 (14.8)	
II	22 (28.2)	13 (26.0)		18 (27.3)	17 (27.4)		13 (19.4)	22 (36.1)	
III	28 (35.9)	18 (36.0)		22 (33.3)	24 (38.7)		29 (43.3)	17 (27.9)	
IV	16 (20.5)	13 (26.0)		16 (24.2)	13 (21.0)		18 (26.9)	11 (18.0)	
*Lymphatic invasion*									
Negative	14 (17.9)	7 (14.0)	.631	10 (15.2)	11 (17.7)	.812	11 (16.4)	10 (16.4)	1
Positive	64 (82.1)	43 (86.0)		56 (84.8)	51 (82.3)		56 (83.6)	51 (83.6)	
*Venous invasion*									
Negative	36 (46.2)	14 (28.0)	.043*	29 (43.9)	21 (33.9)	.279	24 (25.8)	26 (42.6)	.471
Positive	42 (53.8)	36 (72.0)		37 (56.1)	41 (66.1)		43 (64.2)	35 (57.4)	
*Histological grade*									
Well	21 (26.9)	15 (30.0)	.484	18 (27.3)	18 (29.0)	.478	19 (28.4)	17 (27.9)	.988
Moderately	49 (62.8)	33 (66.0)		41 (62.1)	41 (66.1)		43 (64.2)	39 (63.9)	
Others	8 (10.3)	2 (4.0)		7 (10.6)	3 (4.8)		5 (7.5)	5 (8.2)	
*KRAS variant*									
Wild	27 (62.8)	27 (60.0)	.829	30 (66.7)	28 (59.6)	.523	23 (56.1)	31 (66.0)	.385
Mutation	16 (37.2)	18 (40.0)		15 (33.3)	19 (40.4)		18 (43.9)	16 (34.0)	
*BRAF variant*									
Wild	41 (95.3)	44 (97.8)	.612	41 (91.1)	46 (97.9)	.198	40 (97.6)	45 (95.7)	1
Mutation	2 (4.7)	1 (2.2)		4 (8.9)	1 (2.1)		1 (2.4)	2 (4.3)	

Abbreviations: tumoral LOX‐1 score‐L, 0–3; tumoral LOX‐1 score‐H, 4; stromal LOX‐1‐L, ≤534.4 mm^2^; stromal LOX‐1, >534.4/mm^2^; CD8^+^ CTL‐L, ≤103.4/mm^2^; CD8^+^ CTL‐H, >103.4/mm^2^.

* *P* < .05 is considered statistically significant. p‐values were calculated from Fisher's exact tests and chi‐square tests.

Upon combining stromal LOX‐1 and CD8 status, the CRC patients (n = 128) were divided into four groups, that is, stromal LOX‐1‐H/CD8^+^ CTL‐H as H/H (n = 30), stromal LOX‐1‐H/CD8^+^ CTL‐L as H/L (n = 32), stromal LOX‐1‐L/CD8^+^ CTL‐H as L/H (n = 31), and stromal LOX‐1‐L/CD8^+^ CTL‐L as L/L (n = 35). No significant differences were observed in the clinicopathological characteristics among the four groups (Table 2).

**TABLE 2:** Association of combined stromal LOX‐1 and CD8 status with clinicopathological factors in 128 CRC patients

	Stromal LOX‐1/CD8^+^ CTL status				
	H/H	H/L	L/H	L/L	
Factors	n = 30 (%)	n = 32 (%)	n = 31 (%)	n = 35 (%)	*P* value
*Sex*					
Female	12 (40.0)	11 (34.4)	12 (38.7)	13 (37.1)	.972
Male	18 (60.0)	21 (65.6)	19 (61.3)	22 (62.9)	
*Age*					
≤70	20 (66.7)	24 (75.0)	22 (71.0)	20 (57.1)	.442
>70	10 (33.3)	8 (25.0)	9 (29.0)	15 (42.9)	
*T stage*					
T0, T1	4 (13.3)	1 (3.1)	2 (6.5)	6 (17.1)	.178
T2	6 (20.0)	4 (12.5)	2 (6.5)	2 (5.7)	
T3	18 (60.0)	20 (62.5)	19 (61.3)	17 (48.6)	
T4	2 (6.7)	7 (21.9)	8 (25.8)	10 (28.6)	
*N stage*					
N0	18 (60.0)	11 (34.3)	18 (58.1)	15 (42.9)	.06
N1	10 (33.3)	18 (56.3)	7 (22.6)	12 (34.3)	
N2	2 (6.7)	3 (9.4)	6 (19.4)	8 (22.9)	
*M stage*					
M0	26 (86.7)	23 (71.9)	24 (77.4)	26 (74.3)	.528
M1	4 (13.3)	9 (28.1)	7 (22.6)	9 (25.7)	
*TNM stage*					
0	0 (0)	0 (0)	2 (6.5)	1 (2.9)	.084
I	7 (23.3)	1 (3.2)	2 (6.5)	5 (14.3)	
II	9 (30.0)	8 (25.0)	13 (41.9)	5 (14.3)	
III	10 (33.3)	14 (43.8)	7 (22.6)	15 (42.9)	
IV	4 (13.3)	9 (28.1)	7 (22.6)	9 (25.7)	
*Lymphatic invasion*					
Negative	6 (20.0)	5 (15.6)	4 (12.9)	6 (17.1)	.899
Positive	24 (80.0)	27 (84.4)	27 (87.1)	29 (82.9)	
*Venous invasion*					
Negative	13 (43.3)	8 (25.0)	13 (41.9)	16 (45.7)	.302
Positive	17 (56.7)	24 (75.0)	18 (58.1)	19 (54.3)	
*Histological differentiation*					
Well	9 (30.0)	9 (28.1)	8 (25.8)	10 (28.6)	.922
Moderately	19 (63.3)	22 (68.8)	20 (64.5)	21 (60.0)	
Others	2 (6.7)	1 (3.1)	3 (9.7)	4 (11.4)	
*KRAS variant*					
Wild	17 (68.0)	10 (47.6)	14 (63.6)	13 (65.0)	.527
Mutation	8 (32.0)	11 (52.4)	8 (36.4)	7 (35.0)	
*BRAF variant*					
Wild	24 (96.0)	21 (100)	21 (95.5)	19 (95.0)	.895
Mutation	1 (4.0)	0 (0)	1 (4.5)	1 (5.0)	

Abbreviations: stromal LOX‐1‐L, ≤534.4/mm^2^; stromal LOX‐1 score‐H, > 534.4/mm^2^; CD8^+^ CTL‐L, ≤103.4/mm^2^; CD8^+^ CTL‐H, >103.4/mm^2^.

* *P* < .05 is considered significant. *P*‐values were calculated using Chi‐square tests.

### 3.3 Prognostic significance of LOX‐1 and CD8 expression in CRC patients

**FIGURE 3:**
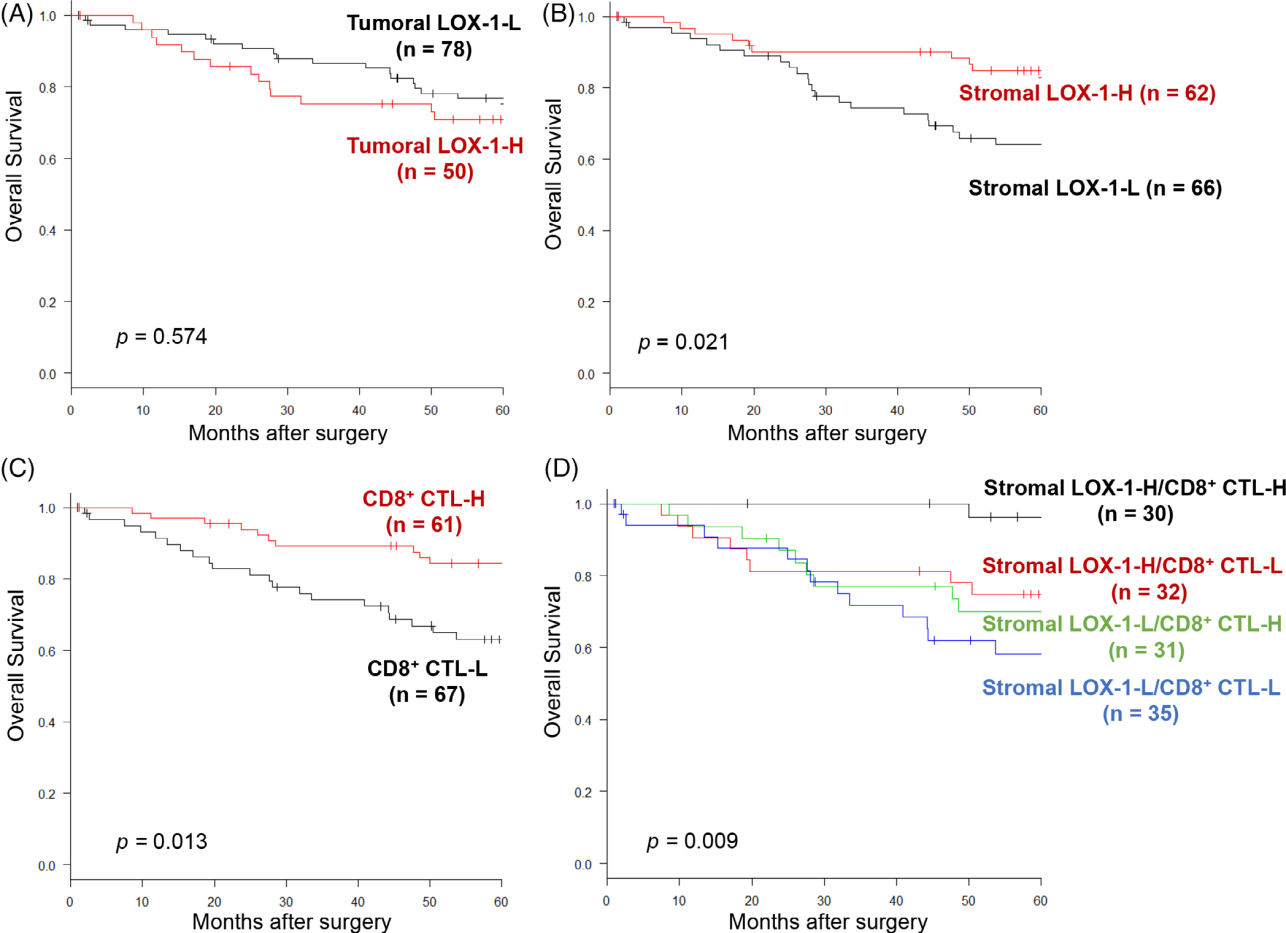
Analysis of overall survival using the Kaplan‐Meier method based on immunological factors. A, High tumoral LOX‐1 expression group (tumoral LOX‐1‐H) vs low tumoral LOX‐1 expression group (tumoral LOX‐1‐L), *P* = .574. B, High stromal LOX‐1 expression group (stromal LOX‐1‐H) vs low stromal LOX‐1 expression group (stromal LOX‐1‐L), *P* = .021. C, High intratumoral CD8^+^ cytotoxic T‐lymphocytes group (CD8^+^ CTL‐H) vs low intratumoral CD8^+^ cytotoxic T‐lymphocytes group (CD8^+^ CTL‐L), *P* = .013. D, Stromal LOX‐1‐H/CD8^+^ CTL‐H vs stromal LOX‐1‐H/CD8^+^ CTL‐L, *P* = .017; stromal LOX‐1‐H/CD8^+^ CTL‐H vs stromal LOX‐1‐L/CD8^+^ CTL‐H, *P* = .021; stromal LOX‐1‐H/CD8^+^ CTL‐H vs stromal LOX‐1‐L/CD8^+^ CTL‐L, *P* < .001; stromal LOX‐1‐H/CD8^+^ CTL‐L vs stromal LOX‐1‐L/CD8^+^ CTL‐H, *P* = .902; stromal LOX‐1‐H/CD8^+^ CTL‐L vs stromal LOX‐1‐L/CD8^+^ CTL‐L, *P* = .204; stromal LOX‐1‐L/CD8^+^ CTL‐H vs stromal LOX‐1‐L/CD8^+^ CTL‐L, *P* = .203. LOX‐1, lectin‐like oxidized low‐density lipoprotein receptor‐1; CD8^+^ CTL, CD8^+^ cytotoxic T‐lymphocytes

Kaplan‐Meier survival curves for the 128 patients with high and low expression of tumoral LOX‐1, stromal LOX‐1, and CD8 are shown in Figure 3. Tumoral LOX‐1 expression did not affect the 5‐year OS rate in patients in the low and high groups (76.7% vs 69.4%; *P* = .574; Figure 3A). However, the stromal LOX‐1‐H group exhibited a higher 5‐year OS rate than the stromal LOX‐1‐L group (84.9% vs 64.1%; *P* = .021; Figure 3B). Moreover, the CD8^+^ CTL‐H group showed a higher OS rate than the CD8^+^ CTL‐L group (82.7% vs 66.6%; *P* = .013; Figure 3C). The 5‐year OS rates were 96.3%, 74.8%, 69.9%, and 58.3% in the stromal LOX‐1‐H/CD8^+^ CTL‐H, LOX‐1‐H/CD8^+^ CTL‐L, LOX‐1‐L/CD8^+^ CTL‐H, and LOX‐1‐L/CD8^+^ CTL‐L groups, respectively (*P* = .009, Figure 3D). The stromal LOX‐1‐L/CD8^+^ CTL‐L group exhibited a poorer prognosis than the stromal LOX‐1‐H/CD8^+^ CTL‐H group (*P* < .001, Figure 3D). Similar results were obtained in the remaining cohort (n = 108), with the exception of stage 0 patients and stage IV patients with unresectable lesions (Figure S1).

The association between clinicopathological parameters and 5‐year OS was examined for the entire cohort using the Cox proportional hazards model (Table 3). Multivariate analysis revealed that the stromal LOX‐1/CD8^+^ CTL status was the most reliable independent prognostic factor for poor OS (HR: 8.55, 95% CI: 1.92‐37.9, *P* = .004).

**TABLE 3:** Univariate and multivariate analyses of the association between various parameters and overall survival

Parameters	Univariate		Multivariate	
	HR (95% CI)	*P* value	HR (95% CI)	*P* value
Sex (female/male)	1.47 (0.72‐3.0)	.28	‐	
Age (≥70/<70)	0.86 (0.42‐1.76)	.692	‐	
TNM stage (0, I, II/III, IV)	4.66 (1.93‐11.2)	<.001*	4.02 (1.34‐12.0)	.012*
Lymphatic invasion (negative/positive)	4.06 (0.97‐16.9)	.054	0.89 (0.15‐5.31)	.903
Venous invasion (negative/positive)	2.37 (1.11‐5.04)	.025*	2.12 (0.94‐4.77)	.06
Histological differentiation (well/others)	1.50 (0.68‐3.31)	.305	‐	
Tumoral LOX‐1 score (H/L)	1.20 (0.62‐2.34)	.574	‐	
Stromal LOX‐1 (H/L)	2.21 (1.10‐4.44)	.024*	‐	
CD8^+^ CTL (H/L)	2.38 (1.17‐4.83)	.016*	‐	
*Stromal LOX‐1/CD8* ^*+*^ *CTL*				
H/H	Reference		Reference	
H/L	5.24 (1.14‐23.9)	.032*	4.06 (0.88‐18.5)	.07
L/H	5.0 (1.08‐23.1)	.039*	6.58 (1.39‐31.0)	.017*
L/L	8.73 (1.99‐38.2)	.004*	8.55 (1.92–37.9)	.004*

Abbreviations: CI, confidence interval; HR, hazard ratio; tumoral LOX‐1 score‐L, 0‐3; tumoral LOX‐1 score‐H, 4; stromal LOX‐1‐L, ≤534.4/mm^2^; stromal LOX‐1‐H, >534.4/mm^2^; CD8^+^ CTL‐L, ≤103.4/mm^2^; CD8^+^ CTL‐H, >103.4/mm^2^.

* *P* < .05 is considered significant.

### 3.4 Correlation between stromal LOX‐1^+^ cells, CD8 ^+^ CTLs, and cytokines expression in the TME

To determine the significance of stromal LOX‐1 expression in the TME, we investigated the correlations between the stromal LOX‐1 status and the expression of the CD8‐derived cytokine IFN‐γ, and the MDSC‐derived cytokine IL‐10, using real‐time RT‐PCR. The expression of the two cytokines did not correlate with stromal LOX‐1 status (Figure S2).

### 3.5 Correlation between stromal LOX‐1^+^ cells, CD8 ^+^ CTLs, and β‐catenin in expression CRC tissues

To assess whether activation of the Wnt/β‐catenin signaling pathway may differ depending on the combined stromal LOX‐1/CD8 status, β‐catenin expression was investigated in 40 CRC patient specimens (20 from stromal LOX‐1‐H/CD8^+^ CTL‐H group and 20 from stromal LOX‐1‐L/CD8^+^ CTL‐L group) using immunohistochemistry. β‐catenin positivity was higher in the stromal LOX‐1‐L/CD8^+^ CTL‐L group than that in the stromal LOX‐1‐H/CD8^+^ CTL‐L group (60% vs 45%; Table 4), but the difference was not significant (*P* = .527).

**TABLE 4:** Comparison of β‐catenin expression between high/high and low/low subsets according to the combined stromal LOX‐1/CD8 status

	Stromal LOX‐1/CD8 + CTL status		
	H/H	L/L	
Factors	n = 20 (%)	n = 20 (%)	*P* value
*β‐catenin*			
Negative	11 (55)	8 (40)	.527
Positive	9 (45)	12 (60)	

Abbreviations: Stromal LOX‐1‐L, ≤ 534.4/mm^2^; stromal LOX‐1 score‐H, > 534.4/mm^2^; CD8 + CTL‐L, ≤ 103.4/mm^2^; CD8 + CTL‐H, > 103.4/mm^2^.

* *P* < .05 is considered significant. *P‐*values were calculated using Fisher's exact test.

### 3.6 The origin of LOX‐1^+^ cells in the TME of CRC tissues

**FIGURE 4:**
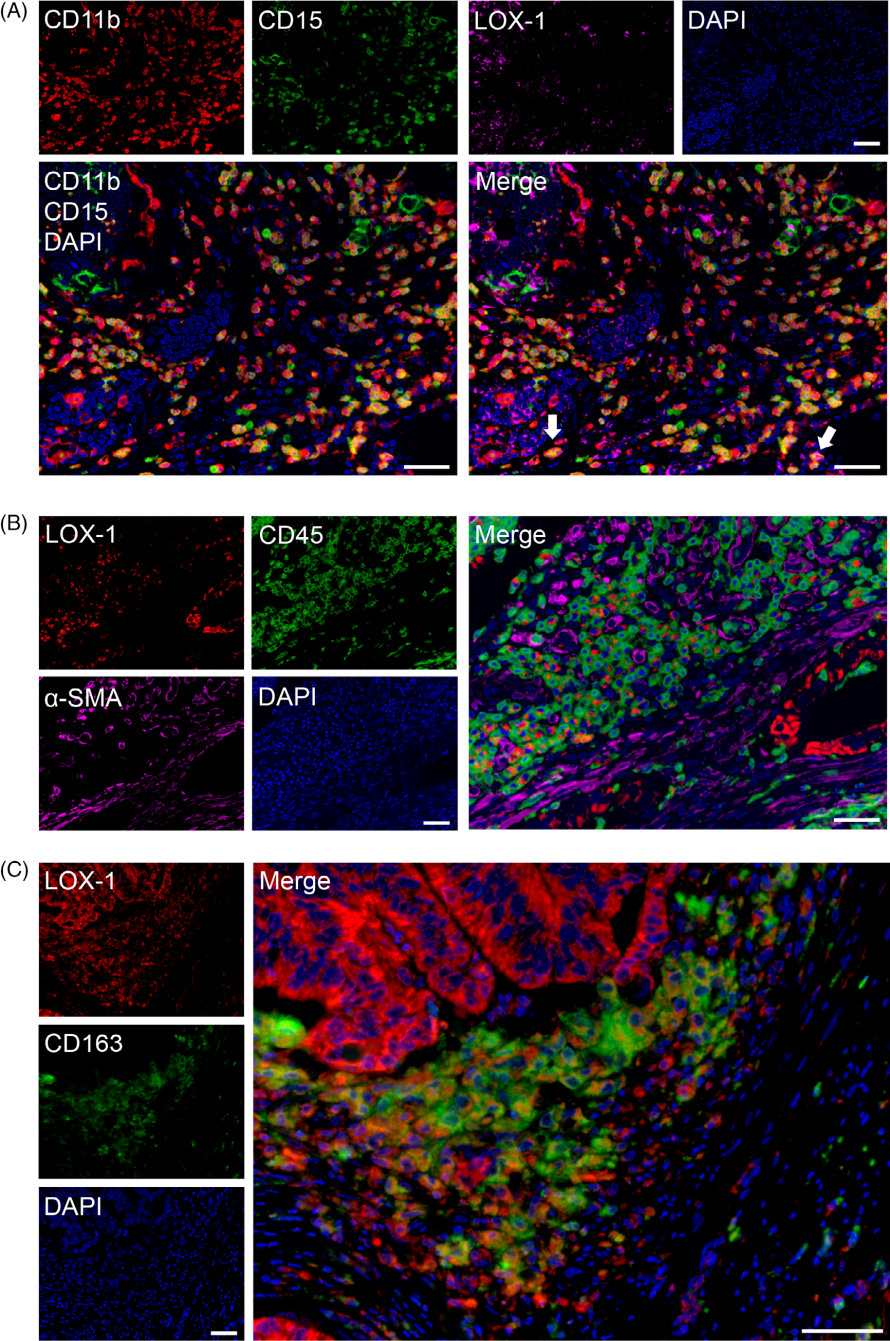
Evaluation of LOX‐1^+^ cells using multiplex immunofluorescence in CRC tissues. A, MDSC markers, CD11b (red), CD15 (green), and LOX‐1 (magenta). Cells co‐expressing CD11b^+^ CD15^+^ are in yellow. Some LOX‐1^+^ stromal cells partially express CD11b^+^ and CD15^+^ cells (white arrows). B, LOX‐1^+^ stromal cells expressed CD45 (green) but did not express α‐SMA (magenta). C, Almost LOX‐1^+^ cells expressed CD163 (green). Nuclei were stained with DAPI (blue). Scale bar = 50 μm. CRC, clinical colorectal cancer; LOX‐1, lectin‐like oxidized low‐density lipoprotein receptor‐1; MDSCs, polymorphonuclear MDSCs

LOX‐1 is a novel marker of MDSCs; however, the expression of stromal LOX‐1 did not correlate with CD8^+^ CTL infiltration and cytokine expression in CRC tissues. Thus, the expression of other MDSC and immune cell surface markers was investigated; these included CD15 and CD11b (MDSC markers), CD45 (pan‐hematopoietic marker), CD3 (pan‐T‐cell marker), CD20 (pan‐B‐cell marker), CD163 (macrophage marker), and α‐SMA (fibroblast marker), to understand the origin of stromal LOX‐1^+^ cells. We analyzed 10 samples from the stromal LOX‐1 high group using triple‐labeling in high‐power fields. LOX‐1^+^ cells were detected in a very small population of typical MDSCs (CD11b^+^ CD15^+^; Figure 4A). All stromal LOX‐1^+^ cells expressed CD45 (Figure 4B), but not CD3, CD20 (Figure S3), or α‐SMA (Figure 4B). Almost stromal cells expressing LOX‐1 also expressed CD163 (Figure 4C).

## 4 DISCUSSION

To the best of our knowledge, this is the first study to analyze the significance of LOX‐1 expression at the immunohistochemistry and spatial levels in the TME of CRC. We showed that CRC patients with low stromal LOX‐1 expression and low levels of CD8^+^ CTL exhibited poor prognosis. Moreover, the combination of low stromal LOX‐1 status and low CD8^+^ CTL counts was the most reliable independent prognostic factor for poor OS. Contrary to previous reports,^25^ no correlation was observed between stromal LOX‐1^+^ cells and immunosuppressive conditions, such as IL‐10 levels, IFN‐γ expression, and density of tumor‐infiltrating CD8^+^ CTLs. Unexpectedly, multiplex fluorescent immunohistochemistry indicated that almost stromal cells expressing LOX‐1 in CRC tissues were CD163^+^ M2 macrophages.

LOX‐1, the main oxidized low‐density lipoprotein receptor, is involved in inflammation, atherosclerosis, and ROS‐ and metabolic disorder‐associated carcinogenesis.^14^ Previous studies reported that LOX‐1 is expressed on tumor cells and is highly expressed in progressive CRC.^18^ Moreover, a high level of serum LOX‐1 is associated with a poor prognosis for patients with CRC.^26^ These studies suggest that LOX‐1 may be an oncogene.^27^ Though these studies involved evaluation of LOX‐1 expression in tumor cells and serum, the sample size was limited. In our current study, we evaluated LOX‐1 expression in the stroma, and the sample size was relatively large. Moreover, stromal LOX‐1‐H was related to a good prognosis in our cohort. From these observations, it was inferred that stromal LOX‐1 functions as an antitumor suppressive factor in CRC.

MDSCs play a key role in tumor immunosuppression; therefore, an abundance of MDSCs in the blood correlates with cancer progression and poor outcome.^28^, ^29^ Herein, we focused on LOX‐1 as a candidate marker for the immunohistochemical detection of MDSCs in tumor tissues. Jiang et al. reported that patients with hepatocellular carcinoma and high levels of LOX‐1^+^ CD15^+^ MDSCs in the peripheral blood exhibit poor OS.^7^ However, our data showed that patients with CRC with high levels of LOX‐1 in the tumor stroma presented longer OS than those with low levels of LOX‐1. Moreover, contrary to previous reports, high levels of LOX‐1 in the tumor stroma did not correlate with the inhibition of CTL‐derived IFN‐γ and induction of MDSC‐derived IL‐10 expression. Our data also demonstrated that LOX‐1^+^ stromal cells partially expressed CD11b and CD15, and almost the stromal cells expressing LOX‐1 were CD163^+^ M2 macrophages. These results indicate that the evaluation of stromal LOX‐1 in clinical CRC samples might be useful for identifying M2 macrophages, but not MDSCs. Reportedly, the stromal infiltration of CD163^+^ M2 macrophages in CRC correlates with improved survival.^30^, ^31^ In contrast, M2 macrophages impart tumor immune tolerance in several cancers excluding CRC.^32^ LOX‐1^+^ cells in stroma were considered as macrophages and constituted a robust prognostic factor for CRC. Further studies are needed to determine whether LOX‐1 expression in tumor tissues is related to the function of LOX‐1^+^ M2 macrophages in the local tumor immunity in CRC tissues.

The stromal LOX‐1‐L/CD8+ CTL‐L group exhibited poor prognosis. Colin et al. had reported that high tumor‐infiltrating lymphocyte counts improve survival in CRC.^33^ Based on immunogenicity and the presence or absence of T‐cells, tumors have recently been classified into immune‐inflamed phenotypes and noninflamed phenotypes (further classified into immune‐excluded and immune‐desert phenotypes).^34^, ^35^ Noninflamed tumors are characterized by low immune reactivity, poor prognosis, and resistance to therapeutics owing to the activation of the localized Wnt/β‐catenin pathway in the TME.^36^, ^37^, ^38^, ^39^ In CRC, activation of Wnt/β‐catenin pathway has been reported to be associated with tumorigenesis and tumor progression.^40^, ^41^ Therefore, we evaluated the relationship between the stromal LOX‐1/CD8 status and Wnt/β‐catenin activation in CRC specimens to understand why the CRC patients with stromal LOX‐1‐L/CD8+ CTL‐L had poorer prognosis compared to other CRC patients. Nuclear accumulation of β‐catenin was identified in 60% of the patients with noninflamed phenotype (stromal LOX‐1‐L/CD8+ CTL‐L) and in 40% of the patients with inflamed phenotype (stromal LOX‐1‐H/CD8+ CTL‐H); however, no significant difference was observed between the inflamed and noninflamed phenotypes. Further analyses using larger cohorts are needed to clarify the fundamental role of the stromal LOX‐1/CD8 status in clinical CRC samples and its association with poor prognosis.

Our study has some limitations. First, limited number of samples were collected from a single institution. Second, we did not perform a functional analysis of LOX‐1 to clarify the correlation between LOX‐1, TME, and tumor immunity in CRC tissues and immune cells.

In conclusion, our data showed that CRC patients with LOX‐1‐L and CD8^+^ CTL‐L phenotypes had a poorer prognosis than patients in other groups. Almost the LOX‐1^+^ cells in CRC stromal tissues were CD163^+^ M2 macrophages. Furthermore, combined LOX‐1/CD8^+^ CTL status was identified as the most important independent prognostic factor for poor OS. LOX‐1 and CD8 status in the tumor stroma may reflect tumor immunogenicity. Accordingly, the combination of LOX‐1^+^ macrophages and CD8^+^ CTL infiltration may be useful in predicting the prognosis of CRC.

## CONFLICT OF INTEREST

The authors declare no conflicts of interest.

## AUTHOR CONTRIBUTIONS

All authors had full access to the data in the study and take responsibility for the integrity of the data and the accuracy of the data analysis. *Study conception and design*, C.K., T.Y., A.K., K.S., H.K., and H.S.; *Acquisition of data*, C.K., T.S., and A.K.; *Analysis and interpretation of data*, All authors; *Drafting of the manuscript*, C.K., T.Y., A.K., K.S., and H.S. All authors reviewed and approved the final manuscript.

## ETHICAL STATEMENT

This study was performed in accordance with the tenets of the Declaration of Helsinki and was approved by the Institutional Review Board for Clinical Research at Gunma University Hospital (Maebashi, Gunma, Japan; Approval No. HS2019‐135). Patient consent was obtained using an opt‐out method.

## Supporting information

**Figure S1** Analysis of overall survival in 108 samples based on immunological parameters using the Kaplan–Meier method.
(a) High stromal LOX‐1 expression group (stromal LOX‐1‐H, > 586.1/mm^2^) vs low stromal LOX‐1 expression group (stromal LOX‐1‐L, ≤ 586.1/mm^2^), *P* = 0.009. (b) High intratumoral CD8^+^ cytotoxic T‐lymphocytes group (CD8^+^ CTL‐H, > 103.4/mm^2^) vs low intratumoral CD8^+^ cytotoxic T‐lymphocytes group (CD8^+^ CTL‐L, ≤ 103.4/mm^2^), *P* = 0.015. (c) Stromal LOX‐1‐H/CD8^+^ CTL‐H vs stromal LOX‐1‐H/CD8^+^ CTL‐L, *P* = 0.126; stromal LOX‐1‐H/CD8^+^ CTL‐H vs stromal LOX‐1‐L/CD8^+^ CTL‐H, *P* = 0.103; stromal LOX‐1‐H/CD8^+^ CTL‐H vs stromal LOX‐1‐L/CD8^+^ CTL‐L, *P* = 0.001; stromal LOX‐1‐H/CD8^+^ CTL‐L vs stromal LOX‐1‐L/CD8^+^ CTL‐H, *P* = 0.897; stromal LOX‐1‐H/CD8^+^ CTL‐L vs stromal LOX‐1‐L/CD8^+^ CTL‐L, *P* = 0.055; stromal LOX‐1‐L/CD8^+^ CTL‐H vs stromal LOX‐1‐L/CD8^+^ CTL‐L, *P* = 0.0786. LOX‐1, lectin‐like oxidized low‐density lipoprotein receptor‐1; CD8^+^ CTL, CD8^+^ cytotoxic T‐lymphocytes

**Figure S2** Relative expression of IFN‐γ and IL‐10 in CRC determined by real‐time RT‐PCR.
(a) IFN‐γ expression in stromal LOX‐1‐H and LOX‐1‐L groups. The data showed no significant difference between the two groups. (b) IL‐10 expression in stromal LOX‐1‐H and LOX‐1‐L groups. The data showed no significant difference between the two groups. Mann–Whitney *U* test was used to evaluate the difference between the two groups. CRC, clinical colorectal cancer; LOX‐1, lectin‐like oxidized low‐density lipoprotein receptor‐1

**Figure S3** Expression pattern of LOX‐1 and lymphocyte makers in CRC tissues.
Lymphocyte cell surface markers. LOX‐1^+^ stromal cells were negative for CD20 (green) and CD3 (magenta). Nuclei stained with DAPI (blue). Scale bar = 50 μm. LOX‐1, lectin‐like oxidized low‐density lipoprotein receptor‐1

## Data Availability

The datasets used or analyzed in this study are available from the corresponding author on reasonable request.
