# Supplementary material for: Low level of stromal lectin‐like oxidized LDL receptor 1 and CD8 + cytotoxic T‐lymphocytes indicate poor prognosis of colorectal cancer
Source: Cancer Rep (Hoboken). 2021 Mar 6;4(4):e1364. doi: 10.1002/cnr2.1364 (PMC8388181; doi:10.1002/cnr2.1364)
Supplement: Supplementary file 1 — Figure S1 Analysis of overall survival in 108 samples based on immunological parameters using the Kaplan–Meier method. (a) High stromal LOX‐1 expression group (stromal LOX‐1‐H, > 586.1/mm2) vs low stromal LOX‐1 expression group (stromal LOX‐1‐L, ≤ 586.1/mm2), P = 0.009. (b) High intratumoral CD8+ cytotoxic T‐lymphocytes group (CD8+ CTL‐H, > 103.4/mm2) vs low intratumoral CD8+ cytotoxic T‐lymphocytes group (CD8+ CTL‐L, ≤ 103.4/mm2), P = 0.015. (c) Stromal LOX‐1‐H/CD8+ CTL‐H vs stromal LOX‐1‐H/CD8+ CTL‐L, P = 0.126; stromal LOX‐1‐H/CD8+ CTL‐H vs stromal LOX‐1‐L/CD8+ CTL‐H, P = 0.103; stromal LOX‐1‐H/CD8+ CTL‐H vs stromal LOX‐1‐L/CD8+ CTL‐L, P = 0.001; stromal LOX‐1‐H/CD8+ CTL‐L vs stromal LOX‐1‐L/CD8+ CTL‐H, P = 0.897; stromal LOX‐1‐H/CD8+ CTL‐L vs stromal LOX‐1‐L/CD8+ CTL‐L, P = 0.055; stromal LOX‐1‐L/CD8+ CTL‐H vs stromal LOX‐1‐L/CD8+ CTL‐L, P = 0.0786. LOX‐1, lectin‐like oxidized low‐density lipoprotein receptor‐1; CD8+ CTL, CD8+ cytotoxic T‐lymphocytes [file CNR2-4-e1364-s002.pptx]

## Slide 1
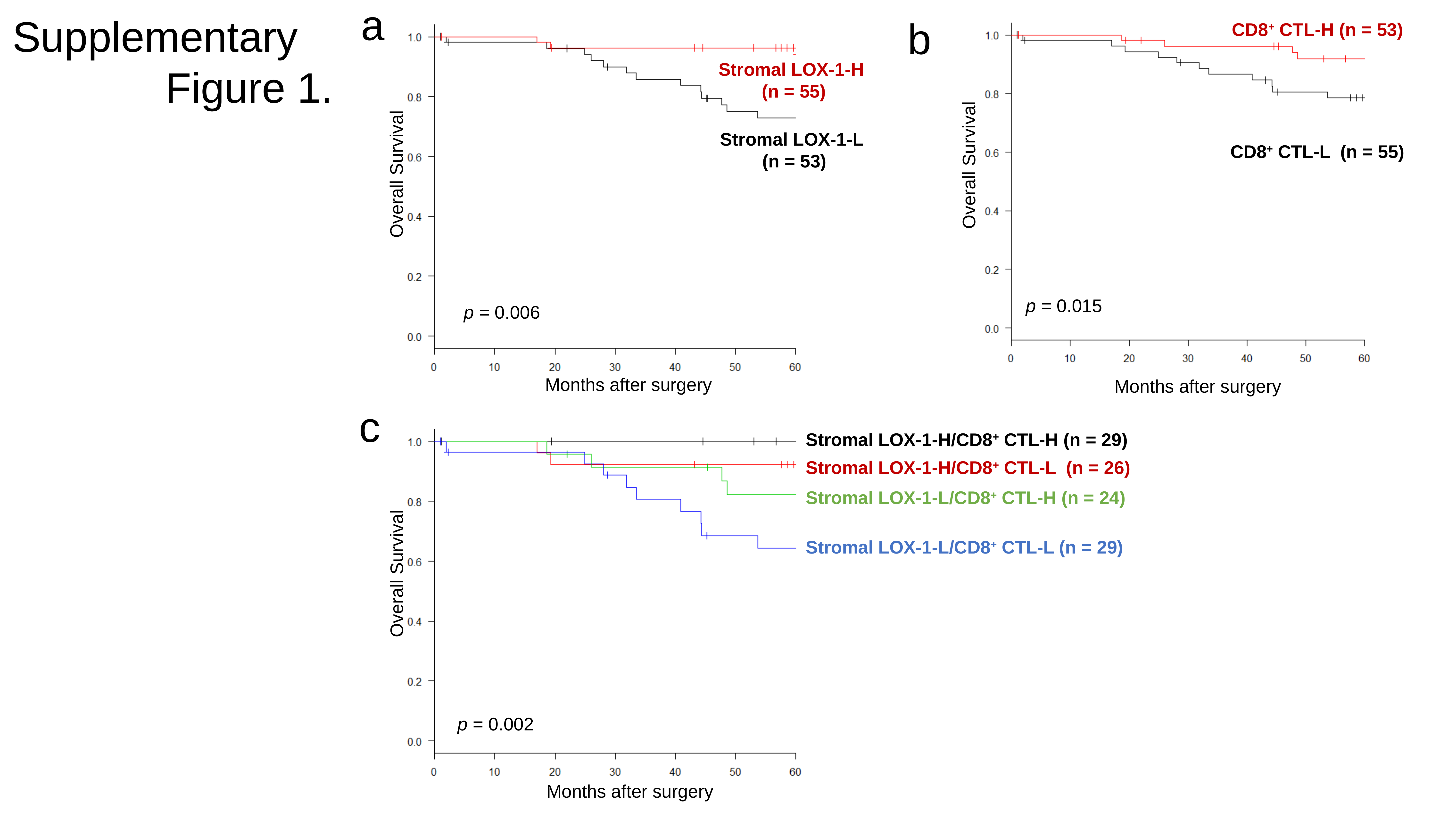

a
Supplementary
 Figure 1.
b
CD8+ CTL-H (n = 53)
CD8+ CTL-L (n = 55)
Overall Survival
Months after surgery
 p = 0.015
Stromal LOX-1-H
(n = 55)
Stromal LOX-1-L
 (n = 53)
Overall Survival
Months after surgery
 p = 0.006
c
Stromal LOX-1-H/CD8+ CTL-H (n = 29)
Stromal LOX-1-H/CD8+ CTL-L (n = 26)
Stromal LOX-1-L/CD8+ CTL-H (n = 24)
Stromal LOX-1-L/CD8+ CTL-L (n = 29)
Overall Survival
Months after surgery
p = 0.002
