# Supplementary material for: Low level of stromal lectin‐like oxidized LDL receptor 1 and CD8 + cytotoxic T‐lymphocytes indicate poor prognosis of colorectal cancer
Source: Cancer Rep (Hoboken). 2021 Mar 6;4(4):e1364. doi: 10.1002/cnr2.1364 (PMC8388181; doi:10.1002/cnr2.1364)
Supplement: Supplementary file 2 — Figure S2 Relative expression of IFN‐γ and IL‐10 in CRC determined by real‐time RT‐PCR. (a) IFN‐γ expression in stromal LOX‐1‐H and LOX‐1‐L groups. The data showed no significant difference between the two groups. (b) IL‐10 expression in stromal LOX‐1‐H and LOX‐1‐L groups. The data showed no significant difference between the two groups. Mann–Whitney U test was used to evaluate the difference between the two groups. CRC, clinical colorectal cancer; LOX‐1, lectin‐like oxidized low‐density lipoprotein receptor‐1 [file CNR2-4-e1364-s001.pptx]

## Slide 1
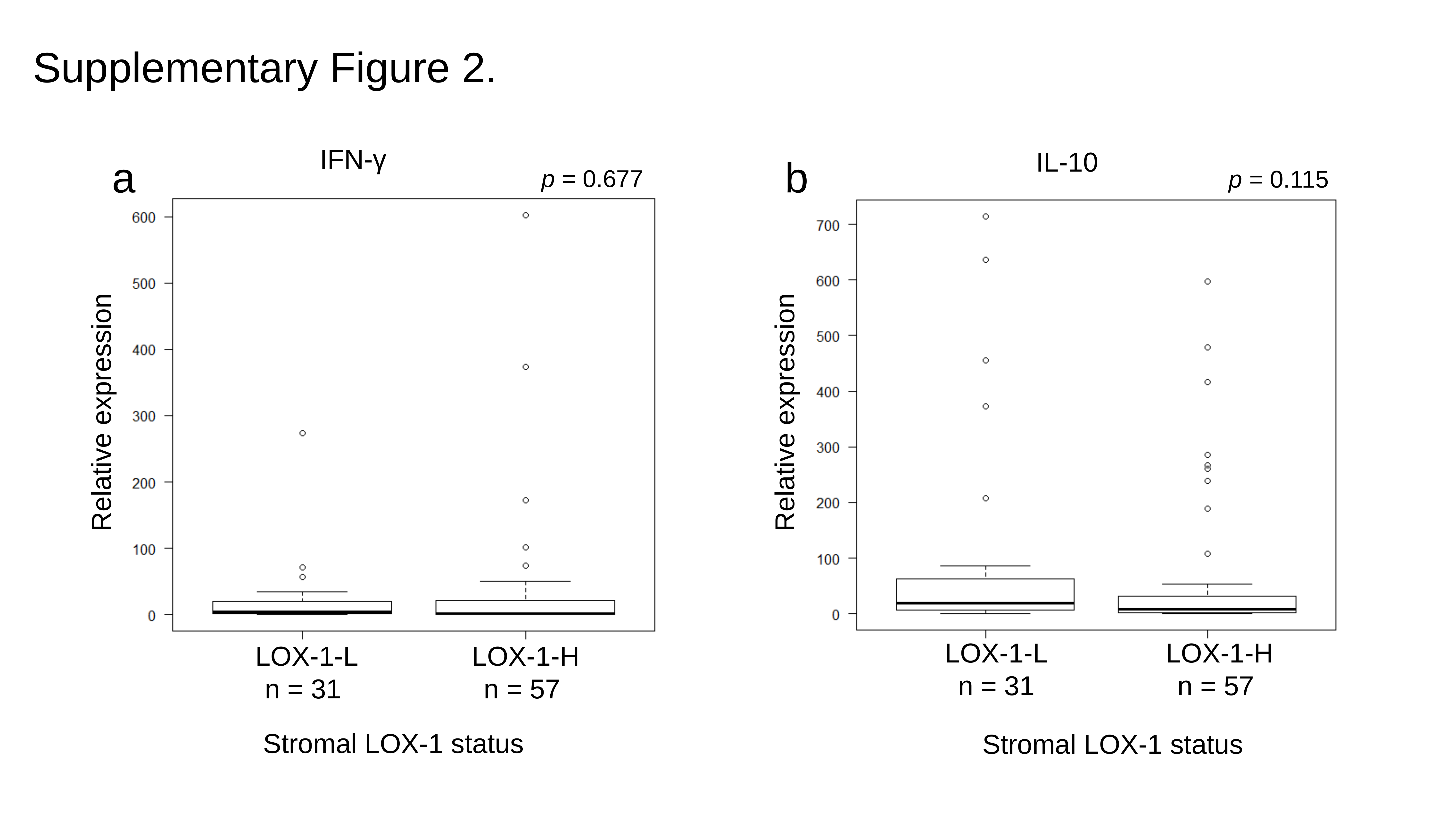

Supplementary Figure 2.
IFN-γ
IL-10
a
b
p = 0.677
p = 0.115
Relative expression
Relative expression
LOX-1-L
n = 31
LOX-1-H
n = 57
LOX-1-L
n = 31
LOX-1-H
n = 57
Stromal LOX-1 status
Stromal LOX-1 status
