# Supplementary figures and images for: Low level of stromal lectin‐like oxidized LDL receptor 1 and CD8 + cytotoxic T‐lymphocytes indicate poor prognosis of colorectal cancer
Source: Cancer Rep (Hoboken). 2021 Mar 6;4(4):e1364. doi: 10.1002/cnr2.1364 (PMC8388181; doi:10.1002/cnr2.1364)

## Slide 1
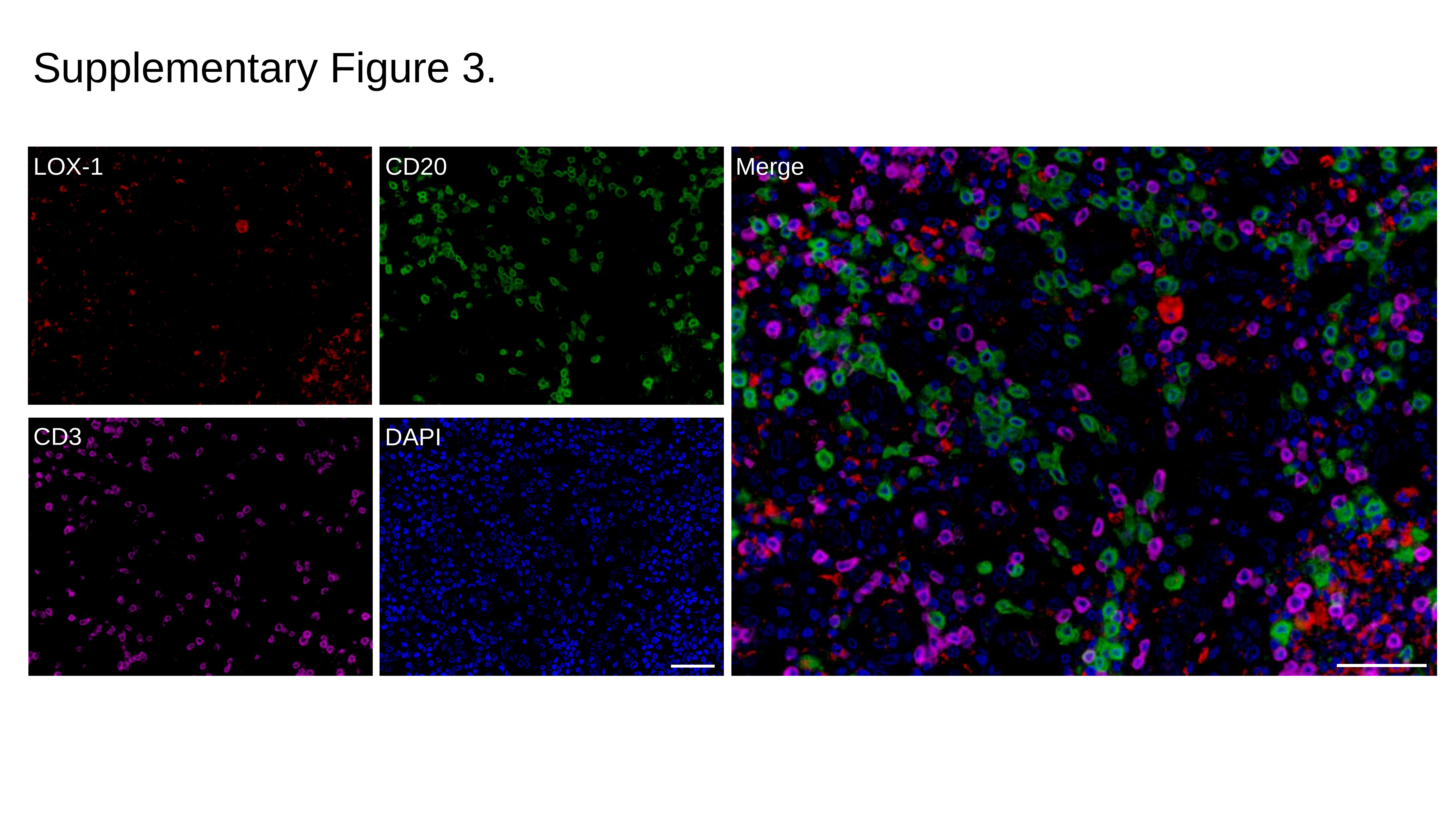

Supplementary Figure 3.
LOX-1
CD20
Merge
CD3
DAPI

Supplement: Supplementary file 3 — Figure S3 Expression pattern of LOX‐1 and lymphocyte makers in CRC tissues. Lymphocyte cell surface markers. LOX‐1+ stromal cells were negative for CD20 (green) and CD3 (magenta). Nuclei stained with DAPI (blue). Scale bar = 50 μm. LOX‐1, lectin‐like oxidized low‐density lipoprotein receptor‐1 [file CNR2-4-e1364-s003.pptx]
